# The Content of Dietary Fibre and Polyphenols in Morphological Parts of Buckwheat (*Fagopyrum tataricum*)

**DOI:** 10.1007/s11130-018-0659-0

**Published:** 2018-02-12

**Authors:** Krzysztof Dziedzic, Danuta Górecka, Artur Szwengiel, Hanna Sulewska, Ivan Kreft, Elżbieta Gujska, Jarosław Walkowiak

**Affiliations:** 1 Institute of Food Technology and Plant Origin, Poznan University of Life Sciences, Wojska Polskiego 31, Poznań, Poland; 2 Department of Pediatric Gastroenterology and Metabolic Diseases, Poznan University of Medical Sciences, Szpitalna 27/33, 60-572 Poznań, Poland; 3 Department of Gastronomy Science and Functional Foods, Poznan University of Life Sciences, Wojska Polskiego 31, 60-624 Poznań, Poland; 4 Department of Agronomy, Poznan University of Life Sciences, Dojazd 11, 60-632 Poznań, Poland; 5 Department of Forest Physiology and Genetics, Slovenian Forestry Institute, Vecna pot 2, 1000 Ljubljana, Slovenia; 6 Department of Commodity Sciences and Food Analysis, University of Warmia and Mazury in Olsztyn, Plac Cieszyński 1, 10-957 Olsztyn, Poland

**Keywords:** Tartary buckwheat, Dietary fibre, Phenolic substances profile, Water extract, Methanol extract

## Abstract

In this report, we presented the profile of polyphenolic substances in flowers, leaves, stalk and roots of *Fagopyrum tataricum* estimated by using RP–UHPLC–ESI-MS equipment (reversed-phase ultra-high-performance liquid chromatography electrospray ionisation mass spectrometry). The neutral detergent fibre, acid detergent fibre, acid detergent lignin, cellulose and hemicellulose were also determined. Flowers, leaves, stalk and roots showed varying levels of dietary fibre and polyphenols. The highest content of neutral and acid detergent fibre were found in the roots (63.92 and 45.45% d.m., respectively) while the most rich in phenolic compounds were flowers (4.8 mg/1 g d.m.). Root and stalk contained the highest level of cellulose, 38.70 and 25.57% d.m., respectively. Among the investigated polyphenolic substances such as: 2,6-dihydroxybenzoic acid, 3,4-dihydroxybenzoic acid, 3,5-dihydroxybenzoic acid, 4-hydrobenzoic acid, caffeic acid, catechin, chlorogenic acid, fagopyrin, ferulic acid, myricetin, gallic acid, isovanilic acid, isovitexin, kaempferol, luteolin, p-coumaric acid, procyanidin B2, quercetin, quercetin 3-D galactoside, rutin, syringic acid and vitexin, we observed that the contents of rutin and chlorogenic acid were the highest. We found some correlation between dietary fibre fractions and individual phenolic substances. The levels of acid detergent fibre (ADF), cellulose and hemicellulose were negatively correlated with isovitexin, kaempferol, vitexin, fagopyrin, caffeic acid and procyanidin B2 content. In this investigation, two solvents (water and methanol) were estimated regarding their extraction efficiency of phenolic compounds. Taking these results into consideration, we recommend using methanol as the extractor to isolate chlorogenic acid, fagopyrin, kaempferol, procyanidin B2, quercetin, quercetin 3-D-galactoside, rutin, vitexin, and water for other investigated polyphenolic substances obtained from *Fagopyrum tataricum*.

**Electronic supplementary material:**

The online version of this article (10.1007/s11130-018-0659-0) contains supplementary material, which is available to authorized users.

## Introduction

Tartary and common buckwheat exhibit different growth behaviour and agrotechnical requirements. In many areas, the trend is to replace common buckwheat and other cereals, which have a lower yielding ability and lack frost tolerance, with tartary buckwheat [1]. Buckwheat is a rich source of bioactive components, such as polyphenolic substances - in particular, rutin, quercetin, soluble and insoluble dietary fibre fractions, valuable vitamins, micronutrients and beneficial proteins, including eight essential amino acids that can be not synthesised by the human body [2]. Because buckwheat grains and buckwheat by-products are a rich source of substances with some beneficial functional properties, this plant has been considered to be a medicinal plant and a material for the production of dietary supplements. It is worth noting that the profile of compounds contained in morphological and anatomical parts of buckwheat depend on different factors, such as the species and the environment [1]. Buckwheat plants contain three classes of flavonoids: flavonols, anthocyanins and C-glucosyl flavones, which were reported to have beneficial properties as food components. These components have antioxidative, hypocholesterolemic and antidiabetic properties [3]. Regarding related health compounds, buckwheat contains fagopyrin. This substance provokes the phototoxic effect known as fagopyrism. The content of fagopyrin is lower in comparison to other antioxidative substances, and probably its content in grain does not have a negative impact on the human health. However, further research needs to be continued [4, 5]. Iminosugars have gained increasing interest due to their high biological activity as glycosidase inhibitor. If used as a dietary supplement or functional food component, D-fagomine may reduce the risks of developing insulin resistance, becoming overweight and suffering from an excess of potentially pathogenic bacteria. Fagomine has also been found to have a potent anti-hyperglycaemic effect [1].

The aim of the study was to evaluate the dietary fibre and polyphenols in morphological parts of buckwheat. Due to insufficient data on the content of dietary fibre and polyphenolic substances in different morphological parts of tartary buckwheat, we decided to investigate into relationships between individual polyphenolic substances and some fractions of dietary fibre. For this purpose, two extractants were used in this study: water and methanol.

## Materials and Method

### Plant Material

Tartary buckwheat (*Fagopyrum tataricum*, Gaertn.) samples were obtained from Breeding Station (harvested 2015, Palikije, Poland). The material originated from tartary buckwheat domestic cultivar from Slovenia (Rangus, Šentjernej) and it consisted all of morphological parts of tartary buckwheat, which include leaves, flowers, stalk and roots. The material was dried and all results were calculated as g/100 g of dry matter (d.m.). These parts were ground by using laboratory grinder (Foss, Sweden).

### Reagents

Thermostable α-amylase (Novozymes, Bagsvaerd, Denmark) was used for digestion of starch. The reagents used to determine the content of neutral detergent fiber (NDF) were: sodium dodecyl sulfate (C_12_H_25_NaO_4_S, Sigma-Aldrich, Saint Louis, USA), neutral disodium versenate dehydrate (C_10_H_14_N_2_Na_2_O_8*_10H_2_O), disodium tetraborate decahydrate (Na_2_B_4_O_7_*10 H_2_O), disodium hydrogen phosphate (Na_2_HPO_4_) and ethylene glycol (Poch, Gliwice, Poland). Reagents used to determine the content of ADF were 1 N sulfuric acid (H_2_SO_4_, 1 N, Poch, Gliwice, Poland) and N-cetyl-N,N,N-trimethylammoniumbromid (C_19_H_42_BrN, Merck, Darmstadt, Germany). Reagents used to determine the content of ADL were: sulfuric acid (72%), and acetone (Poch, Gliwice, Poland). Determination of polyphenolic contents was performed using the reagents and standards of acetonitrile, methanol, 2,6-dihydroxybenzoic acid, 3,4-dihydroxybenzoic acid, 3,5-dihydroxybenzoic acid, 4-hydrobenzoic acid, caffeic acid, catechin, chlorogenic acid, fagopyrin, ferulic acid, myricetin, gallic acid, isovanilic acid, isovitexin, kaempferol, luteolin, *p*-coumaric acid, procyanidin B2, quercetin, quercetin 3-D galactoside, rutin, syringic acid and vitexin, purchased from Sigma Aldrich (Steinheim, Germany).

### The Content of Neutral Detergent Fibre

The content of NDF, consisting of cellulose, hemicellulose and ADL, was determined using the detergent method according to Van Soest and Wine, and Dziedzic et al. [6, 7]. Thermostable α-amylase was used to digest starch. The content of NDF, ADF and ADL was analysed using chemical reagents*.* Hemicellulose (H) content was calculated from the difference between NDF and ADF, while cellulose (C) content was calculated as the difference between ADF and ADL. Analyses were conducted using a Fibertec System M 1020 apparatus by Tecator (Foss, Sweden).

### Extraction and Analysis of Polyphenolic Substances

The extraction was performed by mixing 0.2 g of each sample with 10 mL of solvent (60 °C, 1 h). Methanol, ethanol, acetone and their aqueous solutions are usually used to extract bioactive substances from plants [8–12]. In our study we used two solvents for extraction of polyphenolic substances: methanol (Germany, Sigma Aldrich) and water. Methanol was chosen based on previous studies [8, 13], while water was selected because it is the natural solvent in the human body [14]. The samples (morphological parts of tartary buckwheat) were incubated in methanol or water for 1 h at 60 °C. Subsequently, the samples were centrifuged (4000 x *g*) and filtered (0.45 μm, Millipore). Reversed-phase (C18 column) ultra-high-performance liquid chromatography electrospray ionisation mass spectrometry (RP–UHPLC–ESI-MS) analysis was performed using a Dionex UltiMate 3000 UHPLC (Thermo Fisher Scientific, Sunnyvale, CA, USA) coupled to a Bruker maxis impact ultrahigh resolution orthogonal quadrupole-time-of-flight accelerator (qTOF) equipped with an ESI source and operated in negative-ion mode (Bruker Daltonik, Bremen, Germany). The RP chromatographic separation was achieved with a Kinetex™ 1.7 lm C18 100 A, LC column 100 _ 2.1 mm (phenomenex, Torrance, CA, USA). The ESI-MS settings were as follows: capillary voltage 4500 V, nebulizing gas 1.8 bar, and dry gas 9 l/min at 200°C. The scan range was from mass-to-charge ratio (m/z) 80–1200. The mobile phase was composed of water containing 0.1% formic acid (A) and acetonitrile (B). The flow rate was 0.2 ml/min with a gradient elution of 5–95% B over 20 min. The sample injection volume was 3 μL. The column temperature was set to 40 °C. The ESI-MS system was calibrated using sodium formate cluster ions introduced by loop-injection at the beginning of the LC-MS run. The LC-MS data were processed using Data Analysis 4.1 software (Bruker Daltonik, Bremen, Germany). Molecular ions [M-H] - were extracted from full scan chromatograms and peak areas were integrated. The compounds present in each sample were identified by comparing their retention times with those of standards, and based on molecular mass and structural information from the MS detector.

### Statistical Analysis

Experiments were conducted with three replications. Each value was the mean of three independent trials. One-way analysis of variance (ANOVA) was performed. Hierarchical cluster analysis was carried out using Ward amalgamation rule with the Euclidean distance (d) measure. Tree plots were scaled to a standardized scale (dlink/dmax*100). Non-hierarchical cluster analysis (k-means clustering) was performed to form a grouping of wheat fiber samples. V-fold cross-validation algorithm was used to determine the best number of clusters. Principal component analysis (PCA) technique was used to reduce the dimensionality of data and to present the samples in a new coordinate system. Statistica software, Version 10, StatSoft Inc. (OK, USA) was used to carry out statistical analysis.

## Results and discussion

The investigated morphological parts of buckwheat plants were found to have varying NDF content (Table 1). Roots were found to have the highest dietary fibre content (63.92%), while leaves showed the lowest content (12%). Taking under consideration the level of dietary fibre, the investigated morphological parts of buckwheat can be ordered as follows: roots > stalk > flowers > leaves. The cellulose fraction was dominant in all investigated morphological parts of buckwheat. Root and stalk contained the highest level of cellulose, 38.70 and 25.57% d.m., respectively, while in other investigated parts it ranged from 8.15% d.m. for leaves to 12.86% d.m. in the case of flowers.

**Table 1:** Dietary fibre content in different parts of buckwheat plant (g/100 g d.m. product)

Plant parts	NDF (%)	ADF (%)	ADL (%)	C (%)	H (%)
flowers	20.90^b^	18.95^b^	6.09^c^	12.86^b^	1.95^a^
leaves	12.00^a^	11.35^a^	3.20^a^	8.15^a^	0.64^a^
stalk	37.44^c^	29.68^c^	4.11^b^	25.57^c^	7.77^b^
roots	63.92^d^	45.45^d^	6.76^d^	38.70^d^	18.46^c^

Means with different letters within the columns differ significantly (*p* < 0.05)

**Fig. 1:**
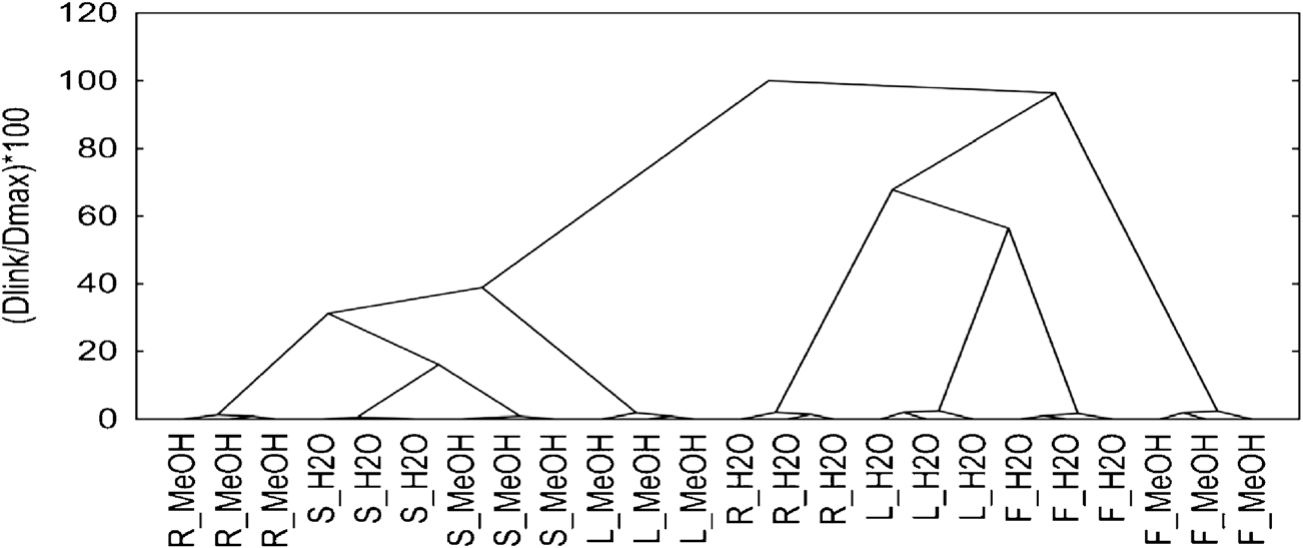
Results of cluster analysis, the variation of biophenolic components in plant parts of buckwheat (F – flower, L – leaf, S – stalk, R – root) based on the extractant used (MeOH – methanol, H_2_O – water). The normalization of scale tree to dlink/dmax / 100 was performed (d – distance, l – linkage, max – maximum of linkage Euclidean distance). Amalgamation rule: Ward’s method, distance metric: Euclidean distances

The roots and leaves constituted the two extremes in terms of the content of the analysed compounds. Intermediate values were found in flowers and stalk, but the content of ADL fraction was the highest in roots. The contents of other dietary fibre fractions in the analysed morphological parts of plants were lower in the flowers than in stalk and roots. The conducted research showed that the fibre content and its properties vary depending on the morphological part of the plant from which the fibre is taken. Furthermore, functional properties of fibre and its chemical structure depend on the function of cell wall properties [15, 16]. Data analysis of methanol and water extracts obtained from buckwheat flowers, leaves, stalks and roots showed the influence of the extracting substance and morphological part of the plant on the profile of investigated phenolic compounds (Fig. 1, Table 2).

**Table 2:** Phenolic compounds content (μg per 1 g d.m.) in different plant parts of buckwheat depending on the extractant used

Variable^*^	Extractant (methanol), plant parts^**^				Extractant (water), plant parts^**^			
	flower	leaf	stalk	root	flower	leaf	stalk	root
2,6-DHBA^W^	3.7^a^	nd	nd	nd	5.5^b^	2.0^a^	nd	nd
3,4- DHBA^W^	186.6^d^	37.3^c^	2.1^a^	6.7^b^	211.8^d^	89.5^c^	6.0^a^	61.1^b^
3,5- DHBA^W^	3.9^a^	9.8^b^	nd	nd	12.0^d^	9.9^c^	1.1^a^	3.8^b^
4-hydroxybenzoic acid^W^	nd	nd	nd	nd	1.4^c^	0.7^a^	nd	1.0^b^
caffeic acid^W^	7.7^b^	10.3^c^	2.9^a^	nd	8.3^b^	18.4^c^	3.1^a^	3.1^a^
catechin^W^	3.0^b^	3.5^b^	1.6^a^	4.7^c^	11.9^d^	9.9^c^	1.1^a^	5.3^b^
chlorogenic acid^M^	1013.4^d^	165.3^c^	102.7^b^	32.7^a^	429.9^d^	203.3^c^	83.7^b^	21.3^a^
fagopyrin^M^	38.2	nd	nd	nd	nd	nd	nd	nd
ferulic acid^W^	nd	nd	nd	nd	4.1^a^	3.2^a^	nd	11.8^b^
gallic acid^W^	nd	2.1^b^	nd	1.6^a^	0.8^a^	7.3^b^	nd	1.4^a^
isovanilic acid^W^	nd	nd	nd	nd	nd	nd	nd	1.6
isovitexin^W^	3.0^c^	1.5^b^	nd	0.5^a^	nd	nd	nd	nd
kaempferol^M^	3.3^c^	nd	nd	nd	nd	2.0^b^	1.4^a^	nd
luteolin^W^	nd	nd	nd	nd	0.9^a^	4.1^b^	nd	nd
*p*-coumaric acid^W^	2.0^c^	4.5^d^	0.8^a^	1.2^b^	8.5^b^	16.9^d^	1.6^a^	12.2^c^
procyanidin B2^M^	439.1^d^	152.3^b^	11.5^a^	263.0^c^	161.2^d^	55.4^b^	6.3^a^	61.6^c^
quercetin^M^	844.7^c^	172.1^b^	2.1^a^	7.2^a^	nd	374.1^a^	352.1^a^	nd
quercetin 3-D-galactoside^M^	10.1^b^	2.3^a^	1.9^a^	9.3^b^	nd	17.8^b^	1.7^a^	nd
rutin^M^	2253.8^b^	2949.3^c^	1255.9^a^	1963.4^b^	nd	576.9^a^	523.8^a^	nd
syringic acid^W^	nd	nd	nd	1.2	nd	nd	nd	5.6
vitexin^M^	42.2^b^	3.3^a^	nd	5.0^a^	nd	nd	nd	nd

^*^Variables with superscript “M” or “W” indicate significant (*p* < 0.05) positive influence of methanol or water as extractant; ^**^Means with different letters within the lines differ significantly (*p* < 0.05), the comparisons were performed separately for methanol and water as extractant, nd- not detected, d.m.- dry matter

Multivariate analysis of variance was used to identify which factor among analysed variables is responsible for the qualitative and quantitative profiles of obtained extracts. We observed a significant (*p* < 0.05) effect of the extractant used, the morphological part of the plant and the interaction between extractant and morphological part of plant on the profile of polyphenolic compounds. Taking into consideration 21 analysed phenolic substances, we identified significantly higher concentration in methanol extracts (Table 2). Many authors reported that the extraction procedure influences the content of selected antioxidative compounds [17, 18]. Masci et al. [19] showed that DPPH and ABTS assay was higher in the case of pomegranate peel water extract in comparison to its ethanol extract. However, the authors obtained opposite results when they investigated pomegranate fruit. This is probably related to the plant matrix and the presence of other compounds.

**Fig. 2:**
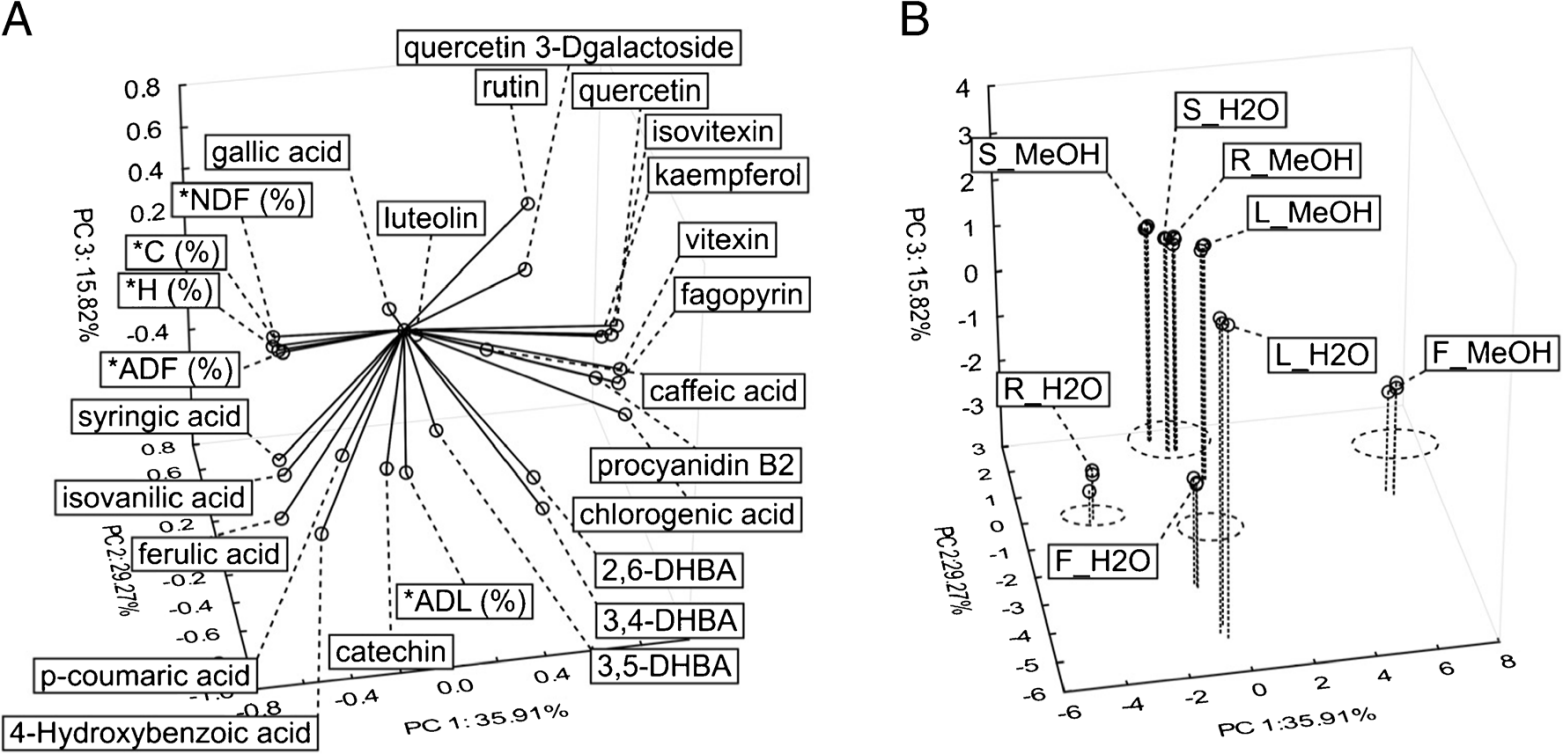
PCA of the loadings plot (A) and the score plot (B) for phenolic components in plant parts of buckwheat (F – flower, L – leaf, S – stalk, R – root) based on the extractant used (MeOH – methanol, H_2_O – water), the supplementary variables were indicated by superscript (*), the principal components were computed using only the active variables

Furthermore, multiple comparisons (Tukey HSD test) of the analysed compounds depending on the extractant used were conducted (Table 2). Methanol used as extracting agent allowed the identification of higher levels of chlorogenic acid, procyanidin B2 and rutin in all morphological parts of plant; vitexin in the case of flowers, leaves and roots, and quercetin and isovitexin only in the case of flowers. Fagopyrin was identified only in flowers using methanol extract. As for rutin, its highest content was observed for leaves, also using methanol extract. There are many reports showing that the efficiency of extraction depends on the material and extractors used during the process and individual antioxidative compounds isolated from parts of plant [3, 18]. The yield of the extraction process depends on a number of factors, such as: the chemical structure of individual isolated components, chemical bonds between plant matrix and isolated compounds, chemical nature of extracted substances and polarity of used solvents: methanol- 5.2; water- 9.0 [11, 19]. No single solvent could extract all flavonoids and polyphenols in buckwheat due to their different polarities and solubilities. Our results suggested that morphological parts of tartary buckwheat plants can be used as a bioactive source of substances for the production of some dietary supplements; however, producers should use proper extractant in relation to the individual polyphenolic substance. We found that all of the analysed dietary fibre fractions were highly correlated with each other; only the correlation coefficient for the ADL fraction with NDF was lower than 0.65 (Table 1S, Online supplementary material). Despite the high correlation between all fractions of dietary fibre we did not observe a similar, uniform tendency as far as the correlation between the individual dietary fibre fractions and the individual polyphenolic substances were concerned. This suggests that not only the extractant, but also the profile of dietary fibre and the content of individual fractions of dietary fibre affect the bioavailability of investigated substances in various morphological parts of buckwheat. Therefore, the entire fibre-antioxidant complex should be considered when explaining DF physiological effects. The morphological parts of plant could be considered a natural source of antioxidant substances for the large intestine bacteria [20]. The supplements produced from morphological parts of buckwheat, rich in dietary fibre and antioxidative substances, can be effectively used against atherosclerosis [10]. For a better understanding of the interaction between analysed values, we carried out a principal component analysis (PCA) - Fig. 2. Relationships of variables were presented in Fig. 2a.

The created model (a system of 3 main components) explains 81% of general variation. Taking into consideration k-means from a V-fold cross test on the scatterplot (Fig. 2b) we separated four groups of variables. The structure of these groups is similar to the results of cluster analysis presented above (Fig. 1). Aqueous extracts of flowers and leaves contained a higher level of DHBA acids as opposed to methanol extracts of stalk, leaves and roots, which were characterised by a high content of rutin. Based on the aforementioned results, we found that the type of extractant did not significantly affect the profile of phenolic compounds in stalk. The mutual relation of vectors (Fig. 2a) suggested that the content of NDF, ADF and also C and H fractions showed a negative correlation with such substances as: isovitexin, kaempferol, vitexin, fagopyrin, caffeic acid and procyanidin B2.

The relation between NDF fraction and all polyphenolic compounds detected in *Fagopyrum tataricum* was calculated. Results of multiple linear regression showed that the extractant was not significant. The generated equation counted only significant relation between independent variable (NDF) and dependent variables (some polyphenolic compounds), *p* < 0.05. The equation presented below (R^2^ = 0.9979) shows standardized coefficients (values in brackets), which allow to perform directly comparisons of individual influence of variables, for example effect of gallic acid is three fold higher than syringic acid - 0.06/0.02 (Table 2S, Online supplementary material).

Equation:

$$ {\displaystyle \begin{array}{l} NDF=-0.69\left(0,10\right) Caffeic\kern0.17em acid+0.09(0.06) gallic\kern0.17em acid+0.46(0.02) syringic\kern0.17em acid\hbox{--} 0.30\\ {}(0.11) luteolin+0.46(0.07) quercetin\;3- Dgalactoside\hbox{--} 0.43(0.08) isovitexin+0.12(0.03)\\ {} procyanidin\;B2\end{array}} $$

The results confirmed that dietary fibre fractions affected the extraction efficiency of phenolic compounds independent of the morphological part of *Fagopyrum tartaricum*. Ajila and Rao [21] showed that a limited free antioxidants present in food products is determined by the physical and chemical interactions between polyphenolic substances and dietary fibre. However, in our study we showed that the efficiency of extraction process depends on individual phenolic substances and the content of dietary fibre.

## Conclusions

The investigated products demonstrated different levels of dietary fibre and polyphenols depending on morphological parts of tartary buckwheat. The level of neutral detergent dietary fibre was the highest in roots. The remaining investigated morphological parts of buckwheat, taking into consideration the content of neutral detergent fibre, can be ordered as follows: stalk>flowers>leaves. It was found that the sum of phenolic compounds obtained from morphological parts of plant using methanol extractant was higher than compounds extracted using water. The highest levels of phenolic compounds were found for flowers. Rutin and chlorogenic acid dominated among polyphenolic substances. We observed that the content of ADF, C and H fractions were negatively correlated with isovitexin, kaempferol, vitexin, fagopyrin, caffeic acid and procyanidin B2. The much higher chlorogenic acid, fagopyrin, kaempferol, procyanidin B2, quercetin, quercetin 3-D-galactoside, rutin and vitexin content in alcoholic extracts compared to aqueous ones confirms the methanol efficiency for *Fagopyrum tataricum.*

## Electronic supplementary material

ESM 1 (PDF 159 kb)

ESM 2 (PDF 145 kb)

## Abbreviations

ADF: Acid detergent fibre
ADL: Acid detergent lignin
C: Cellulose
DHBA: Dihydroxybenzoesic acid
d.m.: Dry matter
H: Hemicellulose
NDF: Neutral detergent fibre
nd: Not detected
PCA: Principal component analysis
