# Supplementary material for: The Content of Dietary Fibre and Polyphenols in Morphological Parts of Buckwheat (Fagopyrum tataricum)
Source: Plant Foods Hum Nutr. 2018 Feb 12;73(1):82–8. doi: 10.1007/s11130-018-0659-0 (PMC5829111; doi:10.1007/s11130-018-0659-0)
Supplement: Supplementary file 1 — (PDF 159 kb) [file 11130_2018_659_MOESM1_ESM.pdf]

1S Correlation coefficients; the presented values are significant at $p < 0.05$

Variable	NDF (%)	ADF (%)	ADL (%)	C (%)	H (%)
NDF (%)	1.0000				
ADF (%)	0.9973	1.0000			
ADL (%)	0.6438	0.6662	1.0000		
C (%)	0.9945	0.9958	0.5956	1.0000	
H (%)	0.9912	0.9788	0.6223	0.9784	1.0000
Correlation coefficients of dietary fiber fractions with components extracted using methanol					
3,5- DHBA	-0.8194	-0.8524	-0.6016	-0.8447	-0.7483
caffeic acid	-0.9680	-0.9768		-0.9825	-0.9387
isovitexin	-0.6139	-0.6048		-0.6732	-0.6220
p-coumaric acid	-0.7195	-0.7626	-0.5792	-0.7507	-0.6316
procyanidin B2			0.6805		
quercetin 3-Dgalactoside			0.9233		
rutin		-0.6001		-0.6156	
syringic acid	0.8840	0.8589	0.6813	0.8421	0.9174
Correlation coefficients of dietary fiber fractions with components extracted using water					
2,6-DHBA	-0.6356	-0.6154		-0.6820	-0.6636
3,5-DHBA	-0.7034	-0.7109		-0.7648	-0.6802
4-hydroxybenzoic acid			0.6506		
caffeic acid	-0.7992	-0.8352	-0.6247	-0.8234	-0.7230
catechin	-0.6021	-0.6153		-0.6746	
chlorogenic acid	-0.7176	-0.6942		-0.7526	-0.7501
ferulic acid	0.6878	0.6572	0.7375	0.6179	0.7337
gallic acid		-0.6010	-0.6051		
isovanilic acid	0.8864	0.8599	0.6841	0.8428	0.9223
kaempferol			-0.9811		
luteolin	-0.7456	-0.7873	-0.6727	-0.7660	-0.6598
quercetin			-0.9623		
quercetin 3-Dgalactoside	-0.6363	-0.6803	-0.7848	-0.6370	

rutin			-0.9688		
syringic acid	0.8858	0.8605	0.6820	0.8436	0.9196
