# Supplementary material for: The Content of Dietary Fibre and Polyphenols in Morphological Parts of Buckwheat (Fagopyrum tataricum)
Source: Plant Foods Hum Nutr. 2018 Feb 12;73(1):82–8. doi: 10.1007/s11130-018-0659-0 (PMC5829111; doi:10.1007/s11130-018-0659-0)
Supplement: Supplementary file 2 — (PDF 145 kb) [file 11130_2018_659_MOESM2_ESM.pdf]

2S Multiple linear regression summary for dependent variable NDF (%)

Variable	b*	Std.Err. of b*	b	Std.Err. of b	p-value
Components extractable with methanol and water (coded as 0), R^2 = 0.9979					
Intercept			0.0000	0.0113	1.0000
caffeic acid	-0.6847	0.0980	-0.6847	0.0980	0.0000
gallic acid	0.0868	0.0573	0.0868	0.0573	0.0492
syringic acid	0.4573	0.0200	0.4573	0.0200	0.0000
luteolin	-0.2990	0.1094	-0.2990	0.1094	0.0147
quercetin 3-Dgalactoside	0.4596	0.0728	0.4596	0.0728	0.0000
isovitexin	-0.4294	0.0770	-0.4294	0.0770	0.0000
procyanidin B2	0.1224	0.0275	0.1224	0.0275	0.0004

b*- standardized coefficients of the regression equation; b- unstandardized coefficients of the regression equation
